# Cardiovascular Anesthesia and Critical Care in the French West Indies: Historical Evolution and Current Prospects

**DOI:** 10.3390/jcm14020459

**Published:** 2025-01-13

**Authors:** Christian Isetta, François Barbotin-Larrieu, Sylvain Massias, Diae El Manser, Adrien Koeltz, Patricia Shri Balram Christophe, Mohamed Soualhi, Marc Licker

**Affiliations:** 1 Department of Cardiovascular & Thoracic Anaesthesia and Critical Care, University Hospital of Martinique, F-97200 Fort de France, France; isetta.christian0774@orange.fr (C.I.); francois.barbotin-larrieu@chu-martinique.fr (F.B.-L.); sylvain.massias@chu-martinique.fr (S.M.); diae.elmanser@chu-martinique.fr (D.E.M.); adrien.koeltz@chu-martinique.fr (A.K.); shri.balram@chu-martinique.fr (P.S.B.C.); mohamed.soualhi@chu-martinique.fr (M.S.); 2 Faculty of Medicine, University of Geneva, CH-1206 Geneva, Switzerland

**Keywords:** cardiopulmonary bypass, Caribbean territories, cardiac surgery, interventional cardiology, extracorporeal membrane oxygenation: air ambulance transportation

## Abstract

Anesthesiology, the medical specialty that deals with the management of vital functions in patients undergoing surgery, has played an important role in the successful development of cardiac interventions worldwide. Tracing the historical roots of cardiac anesthesia and critical care from its inception in the late 1950s, a paradigm shift in perioperative care has been driven by a better understanding of the mechanisms of organ dysfunction in stressful conditions and technological advances regarding surgical approach, patient monitoring, and organ protection. Although progress in cardiac anesthesia and critical care lagged a little behind in Caribbean territories, successful achievements have been accomplished over the last forty years. Compared with Western countries, the greater prevalence of obesity, diabetes mellitus, and hypertension as well as specific diseases such as cardiac amyloidosis, sickle cell anemia, rheumatic heart disease, and tropical infections may reduce a patient’s physiologic reserve and increase the operative risk among the multi-ethnic population living in the French West Indies and Guiana. So far, cardiac anesthesiologists at the University Hospital of Martinique have demonstrated their abilities in implementing evidence-based clinical care processes and adaptating to efficiently working in a complex environment interacting with multiple partners. Attracting specialized physicians in dedicated cardiac surgical centers and the creation of a regional health network supported by governmental authorities, insurance companies, and charitable organizations are necessary to solve the unmet needs for invasive cardiac treatments in the Caribbean region.

## 1. Introduction

### 1.1. Cardiovascular and Thoracic Surgeries

General anesthesia emerged as a new medical discipline in 1846 with the demonstration of the sedative, analgesic, and relaxant effects of the «laughing gas», nitric oxide [1]. The history of cardiothoracic anesthesia is more recent and was initially driven by the surgical challenges to correct (or palliate) congenital heart diseases and maintain gas exchange during lung interventions [2].

Cardiac surgery traces its origin back to the first closed-chest mitral commissurotomy (in 1923, Henry Souttar) and ligation of a patent ductus arteriosus (in 1938, Robert Gross) [3]. In thoracic surgery, the threatening condition of pneumothorax was overcome by mastering the upper airways with tracheal intubation and by introducing mechanical ventilation and lung isolation techniques that allowed invasive treatment of pulmonary tuberculosis and cancer [4]. In the early 1950s, the advent of cardiopulmonary bypass along with myocardial and cerebral protection techniques paved the way for the development of open-heart surgery, coronary artery bypass, and treatment of major vessel diseases allowing the surgeon to operate on an arrested heart and almost bloodless field [5].

In the 1960s, European cardiac surgery was initiated by a few pioneers in France (Charles Dubost and Christian Cabrol), in the UK (Russell Brock and Thomas Holmes Sellors), in Sweden (Clarence Crafoord), in Germany (Ernst Derra and Rudolf Zenker), in Italy (Achille Mario Dogliotti), in Russia (Alexander Bakulev), and in the Netherlands (Gerard Brom), to mention only a few [6]. Yet, implementation of these innovative treatments were slower than in United States (US) due to the aftermath of the Second World War and a lack of financial support for scientific research [6].

On 12 May 1986, the European Association of Cardio-Thoracic Surgery (EACTS) was officially registered in Paris and in July 1987, the first issue of the European Journal of Cardio-Thoracic Surgery (EJCTS) was launched to describe new operative techniques and share scientific findings among clinicians. The Caribbean islands did not lag far behind the Western world since Cuba established cardiac surgery in 1951 (Havana, Instituto de Cirugía Cardiovascular y Torácica), followed by Jamaica in 1968 (Kingston) and Martinique (Fort de France) and Puerto Rico in the late eighties [7].

### 1.2. Cardiothoracic Anaesthesia

Cardiac and thoracic anesthesia first evolved in the shadow of cardiothoracic surgery and became new subspecialties by incorporating technological and procedural innovations making patient care safer and improving surgical outcomes (e.g., pulmonary artery catheterization, echocardiography, and blood management) [8]. Concomitantly, a few anesthesiologists laid the foundation of intensive care units (ICUs) during the polio epidemic by treating respiratory failure with mechanical ventilation [9]. After cardiothoracic operations, it is was quickly recognized that the management of early complications (i.e., bleeding, respiratory failure, acute kidney injury, low cardiac output syndrome, and sepsis) was just as important for a successful outcome as the repair of cardiac disorders. Multidisciplinary involvement of surgeons, dedicated anesthesiologists/ICU physicians, cardiologists, physiotherapists as well as ICU and operating room nurses was key to the blossoming of cardiothoracic clinical care and training programs [10].

In North America, the Society of Cardiac Anesthesia (SCA) was created in 1978. The European Association of Cardio-Thoracic Anaesthesia (EACTA) was founded in 1985 and was renamed the Association of Cardio-Thoracic Anesthesia and Intensive Care (EACTAIC) to incorporate the full spectrum of perioperative care. In France, the Cardiothoracic and Vascular Anesthesia and Intensive Care Association emerged in 2002 from the French Society of Anesthesia and Reanimation. Of note, in many European countries, anesthesia and critical care belong to the same department and their physicians are board-certified in these subspecialties, sharing their clinical activities in preoperative assessment clinics, operating rooms, and ICUs [11].

Herein, we describe the clinical specificities of the Caribbean population and key steps that led to the creation of the Department of Cardiac Anesthesia and Critical Care at the University Hospital of Martinique (UHM) along with its contribution to the health care network in the region. In addition to individual testimonies of local health care workers, a literature review was performed using PubMed/MEDLINE and Google Scholar databases to identify articles on the history, current state of practice, and advances in cardiovascular surgery in the French Indies.

## 2. Characteristics of the Population

### 2.1. Socio-Geographical Aspects

**Figure 1:**
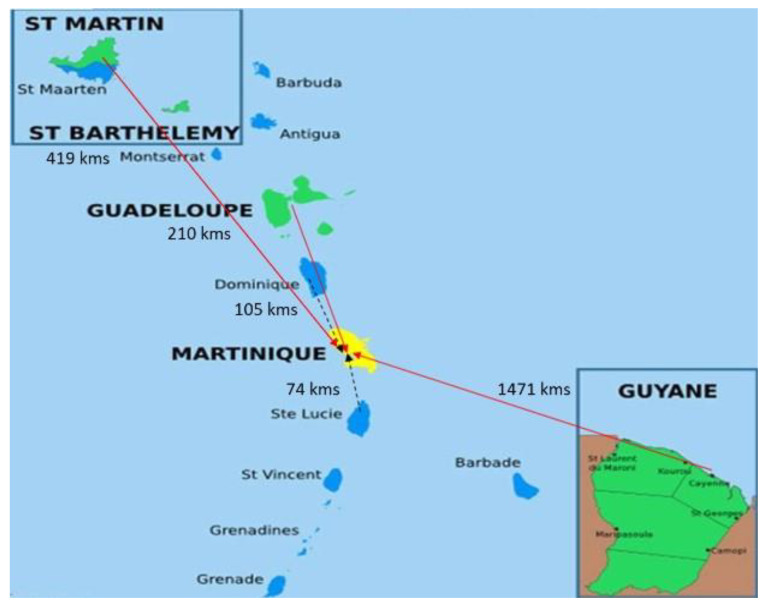
The Caribbean cardiovascular network organized with the University Hospital in Martinique (Fort de France) and hospitals located in other French territories (St. Martin, Guadeloupe, and French Guiana) and some neighboring islands (St. Lucie and Dominique). The distance between these remote locations and Fort de France is expressed in km.

The Caribbean archipelago extends almost 3000 km from Cuba to Trinidad and Tobago, enclosing the Caribbean Sea with three major islands groupings: the Lesser Antilles, the Greater Antilles, and the Bahamas. The current population of more than 46 million spreads over more than 700 islands organized into 30 territories with a wide range of gross national income per capita (i.e., from USD 31,150 in the Bahamas to USD 1610 in Haiti) [12]. About 1.3 million individuals live in the French overseas territories (Martinique, Guadeloupe, St. Martin, and French Guiana; Figure 1) and include a dominant proportion of Afro-Caribbean and Caribs or Kalinago people, in addition to a large diversity of other ethnic groups due to sustained flows of immigration and descendants of French settlers [13]. All inhabitants in the French overseas territories benefit from free access to the national health care system. In the French Indies and Guiana, a regional health system program relies on a network of public hospitals, with a few additional private clinics and home care offices where treatments are free or low-cost. Whenever procedures and treatments cannot be provided in overseas territories, the patient is given the possibility of being transferred and treated on the mainland free of charge.

### 2.2. Health Issues in Caribbean Countries

#### 2.2.1. Life Expectancy and Cardiovascular Risk Factors

Compared with French Metropolitan departments, life expectancy is quite similar in Martinique (women 83.8 and men 78.2 years), Guadeloupe (women 84.3 and men 76.9 years), and French Guiana (women 81.9 and men 76.1 years). The lower tobacco consumption compared to French Metropolitan departments reduces the development of cancer and cardiovascular diseases. In contrast, a sedentary life style and dietary habits result in a higher prevalence of obesity (mainland France: 16% in women and 15% in men; Guadeloupe: 23% and 17%; Martinique: 23% and 21%; and French Guiana: 23% and 15%), arterial hypertension (20% in Metropolitan France, 30% in Guadeloupe, 31.5% in Martinique, and 22.7% in Guiana), and diabetes mellitus (5.7% in Metropolitan France, 12% in Guadeloupe, 11.5% in Martinique, and 11.6% in Guiana) [14]. In addition, Afro-Caribbean patients more frequently experience symptomatic peripheral arteriopathy and present higher prevalences of chronic kidney disease and stroke compared to Caucasians [15,16]. Given the higher burden of atherosclerotic disease, silent myocardial ischemia may lead to missed or delayed diagnosis of myocardial infarct, sudden death, and the development of heart failure [17]. Moreover, popular beliefs in ancestral remedies and distrust in the health system can result in poor adherence to and compliance with prescribed treatments [18]. Up to 50% of Caribbean people regularly use complementary and alternative medicine, mainly herbal remedies, that may result in delayed diagnosis and proper treatment [19].

#### 2.2.2. Infections and Rheumatic Heart Disease

As in other tropical regions, seasonal outbreaks of Dengue fever are common and sporadic cases of typhoid infection, leptospirosis, and tuberculosis develop in the Caribbean territories [20]. Regarding human immunodeficiency virus (HIV) infections, the incidence is much higher among residents from French Guiana (38.6/100,000), Guadeloupe (7.6/100,000), and Martinique (6.7/100,000) than in mainland France (2.3/100,000) [21]. Accordingly, close attention should be paid to patient history before major surgery and preoperative biological assessments should include serological testing of high-risk individuals and control of viral loads in treated patients.

Rheumatic heart disease (RHD) and infective endocarditis (IE) represent other important health issues in the Caribbean region. Based on data from the Global Burden of Disease (GBD) Study 2019, the mean age-standardized prevalence rate of RHD in the Caribbean region is much higher than in Western Europe (795 vs. 41/100,000, respectively) and it will continue to increase from 2020 to 2030 (+0.10% annually), whereas the prevalence of heart failure will stabilize and the RHD-associated mortality is expected to decline [22,23].

In parallel to the increasing burden of RHD, the age-standardized incidence of IE has increased worldwide between 1990 and 2019, from 9.9 to 13.3 per 100’000 individuals, with the highest numbers being reported in the Caribbean territories (increase from 11.1 to 18.7 per 100,000) [24]. Despite diagnostic and therapeutic improvements, IE remains associated with in-hospital deaths of 22% and a 5-year survival of 60% [25].

From 2001 to 2013, 201 patients with IE were admitted and treated at the UHM; 47 (23%) were transferred from Guadeloupe, 25 (12.4%) from another hospital in Martinique, and 16 (8%) from French Guiana [26]. Compared with similar cases from Western countries, patients with IE at the UHM were younger and presented more frequently with negative blood cultures and less commonly with streptococci and enterococci strains [26]. Cardiac surgery was performed in 107 patients (53%) and was associated with a lower mortality compared with medical treatment (odds ratio of 0.21 and 95% confidence interval of 0.1 to 0.46).

#### 2.2.3. Specific Non-Communicable Diseases

Among people of West African descent, transthyretin cardiac amyloidosis (TTR-CA) with its two main forms, wild and hereditary types, is increasingly recognized as an important cause of heart failure, accounting for nearly a third of diastolic heart failure [27]. Almost 4% of Afro-Caribbeans present a mutation linked to the development of cardiac amyloidosis [28]. Before cardiovascular operations, TTR-CA should be screened in patients with heart failure, syncope, and ECG or echocardiographic markers of ventricular hypertrophy.

Drepanocytosis or sickle cell anemia (SCA) affects 0.2 to 0.3% of Afro-American and Caribbean people with its homozygous form (Hb-SS) and 7 to 8% of individuals with the heterozygous form (Hb-SA) [29]. Under regional hypoxic conditions triggered by stressful conditions (i.e., surgery, hypothermia, hypovolemia, and infection), red blood cells undergo morphological changes due to HbS polymerization resulting in increased blood viscosity, arteriolar vasoconstriction, and endothelial damage that promote low microvascular flow and vascular occlusion [30]. Lower survival and poor quality of life are the consequences of recurrent vaso-occlusive crises leading to congestive heart failure, stroke, thromboembolism, kidney disease, and pulmonary lesions [31,32]. Interestingly, a propensity score-matched analysis of cases recorded in the Society of Thoracic Surgeons database indicated similar operative mortality in patients with SCA and their matched controls [33]. These data emphasize the importance of preoperative preparation involving hematologists and intra- and postoperative monitoring of temperature and oxygenation parameters (i.e., hemoglobin and near-infrared spectroscopy) when these patients undergo cardiac surgery [34,35].

Altogether, Caribbean patients who undergo major intervention are expected to present higher cardiovascular operative risks given the increased prevalence of obesity, diabetes mellitus, renal insufficiency, vascular diseases, cardiac amyloidosis, and infectious diseases (Table 1). Indeed, despite their younger age, the burden of the aforementioned metabolic and cardiovascular disorders and infections may reduce a patient’s physiologic reserve and increase the risk of postoperative organ dysfunction. Since the EuroSCORE does not include these potential risk factors (i.e., morbid obesity, noninsulin-dependent diabetes, and race/ethnicity), the operative risk could be underestimated in the Caribbean population [36].

## 3. Development of Cardiac Surgery, Anesthesia, and Critical Care in Martinique

### 3.1. Centralization of Medical Care, from a General Hospital to an Academic Center

During the 19th century, the first military hospital in Fort de France was transformed into a public care center with religious nursing staff to treat victims of trauma and patients afflicted with infectious or chronic illnesses. In the mid-20th century, the French government strongly supported a health care network in all overseas departments. Therefore, major work was undertaken in Fort de France that led to the opening of new operating rooms and the maternity ward was moved to another site.

**Figure 2:**
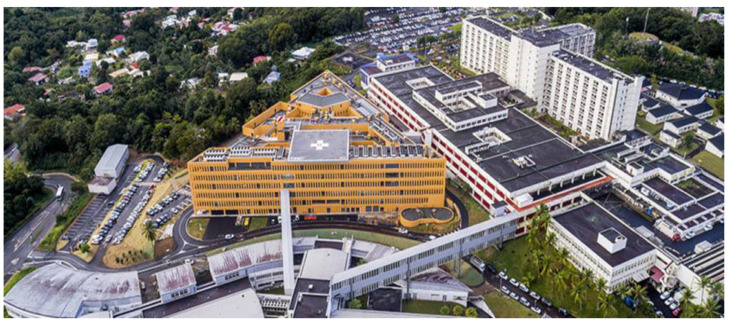
Aerial view of Pierre Zobda Quitman Hospital and Mothers–Women–Children Hospital, the main site of the University Hospital of Martinique in Fort de France.

On 13 March 1984, the Hopital La Meynard became the main regional hospital center in Martinique to provide 24 h emergency services and a full range of interventions (except transplantation) as well as medical, nursing, and rehabilitation activities. In 2013, the 1600-bed institution was renamed “University Hospital of Martinique” (UHM) and was reorganized into three main sites (Adult, Women, and Children Hospitals) and six peripheral sites, acting as a real Caribbean Center for Clinical Care as well as teaching and training for all health care providers (Figure 2).

### 3.2. Cardiac Surgery in Fort de France

**Figure 3:**
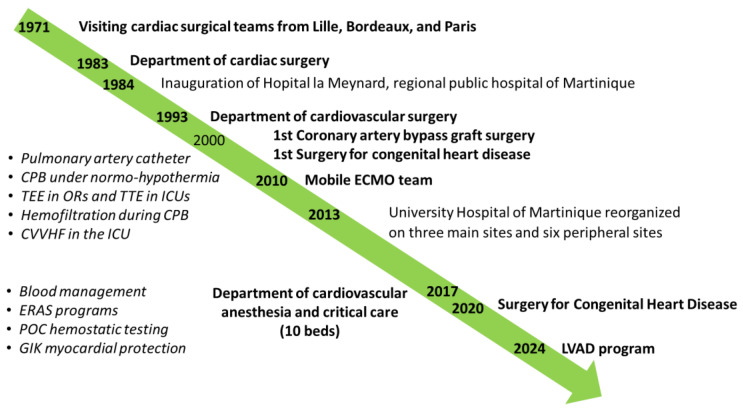
Illustration of key milestones in the development of cardiovascular surgery associated with the emergence of cardiac anesthesia and critical care at the University Hospital of Martinique. CVVHF, continuous veno-venous hemofiltration; CPB, cardiopulmonary bypass; ERAS, enhanced recovery after surgery; GIK, glucose–insulin–potassium; ICU, intensive care; LVAD, left ventricular assist device; OR, operating room; POC, point-of-care; TEE, transesophageal echocardiography; TTE, transthoracic echocardiography.

In the early 1970s, cardiac surgical sessions were periodically conducted over one week by visiting teams from the university hospitals of Lille, Bordeaux, and Paris. Since local cardiac surgeons, perfusionists, and anesthesiologists/intensivists had all been trained in large reference cardiac centers in mainland France, both elective and emergency procedures were performed from its inception, according to international standards of care. Under the leadership of Francis Fontan, chief of the Cardiothoracic Surgical Department at the University Hospital in Bordeaux, a close collaboration was initiated with the UHM to consolidate formal training and enhance skills and competencies of the local medical and nursing teams (Figure 3). In 1983, the department of cardiac surgery was created, being extended to include vascular surgery in 1993 and thoracic surgery in 2005.

In the late 1990s, François Roques, a long-time chief of the department of CVT surgery and current medical director at the UHM, led seminal scientific works in collecting perioperative information and analyzing large databases to establish the EuroSCORE that is currently applied to predict the risk of mortality after cardiac procedures [36]. This example highlights the opportunities to participate in major scientific projects through an international scientific network, even when based in remote locations.

Over the last 3 decades, cardiovascular and thoracic (CVT) surgery at UHM has been modernized with the latest technologies (i.e., CT scan, magnetic resonance imaging, nuclear scintigraphy, and hyperbaric chamber), the opening of a hybrid operating room, and implementing modern surgical approaches (i.e., minimally invasive surgery, off-pump coronary bypass graft, and endovascular surgery).

Presently, the department of CVT surgery at the UHM includes three board-certified cardiac surgeons, two vascular surgeons, and one thoracic surgeon who annually perform on average 350 cardiac operations, 1500 vascular interventions, and 200 thoracic surgeries, mainly video-thoracoscopic surgeries.

Approximately 40% of all operative cases are transferred from other islands (Guadeloupe, St. Martin, Dominica, and St Lucia) and French Guiana where preoperative investigations are completed before admission to the UHM. On a monthly basis, cardiac surgeons conduct on-site consultations in Pointe à Pitre (Guadeloupe) and in Cayenne (French Guiana), to evaluate and select surgical candidates, in cooperation with local cardiology teams. Flexibility in planning and managing the weekly elective operative program is required in order to integrate the high burden of urgent/emergency cases (40% of the total case load) such as acute aortic dissection, severe decompensated valvular disease, acute coronary syndrome, and acute infective endocarditis. Transportation of these acute surgical cases is coordinated and managed by the medical emergency team of the UHM by ambulance (inside the islands), by helicopter (from neighboring islands), and/or by a military/commercial flight (from French Guiana or St. Martin).

### 3.3. ECMO Team in French West Indies

**Figure 4:**
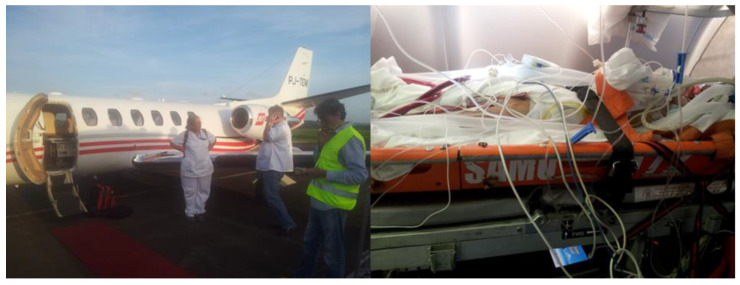
Air ambulance and ECMO mobile team with a critically ill patient.

Since 2010, veno-venous and venoarterial extracorporeal membrane oxygenation (VV and VA ECMO) are provided in the general and cardiac ICUs in Fort de France to support patients with respiratory, circulatory, and cardiopulmonary failure. Cardiac surgeons, perfusionists, and emergency and anesthesia/ICU physicians have set up an ECMO mobile team (Figure 4) [37]. Information related to the patient’s condition is shared through phone calls and an internet network by all physicians working in emergency medical departments, acute cardiology care, and general and cardiac ICUs in the aforementioned Caribbean territories. All cases are discussed with cardiac surgeons and ICU physicians who provide advice for the optimization of pharmacological treatment and to confirm the decision for VV or VA ECMO implantation and return of these critically ill patients to the cardiac ICU at the UHM. Some patients who are suitable candidates for heart transplantation are secondarily transferred with/without ECMO support to mainland France. Over a 9-year period (2011–2019), 26 patients under ECMO were transferred on a commercial flight to the Hôpital La Pitié-Salpétrière in Paris (7260 km and 14 h flight time) for further treatment (7 transplantations, 9 ventricular assist devices [LVADs], and 10 medical treatments) [38].

#### Cardiovascular and Thoracic Anesthesia and Critical Care

**Figure 5:**
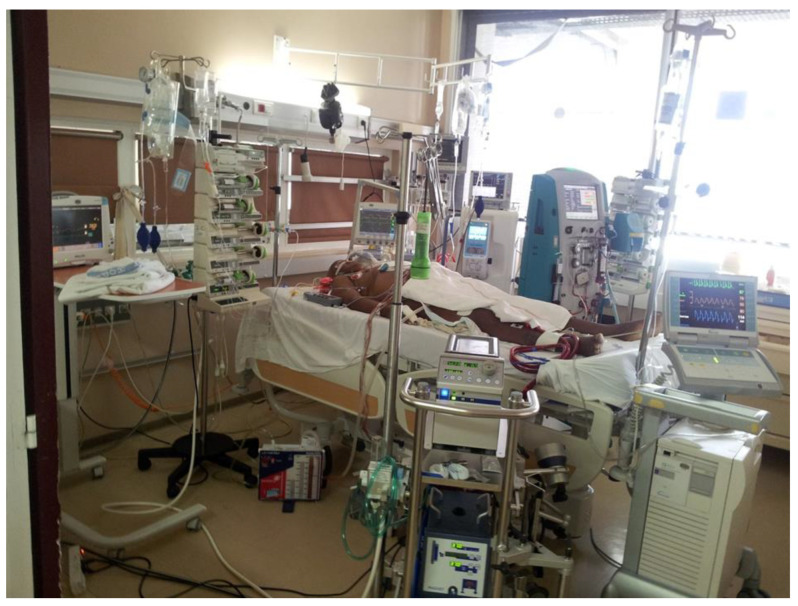
Intensive care station equipped with modern ventilators, advanced hemodynamic monitoring, continuous veno-venous hemofiltration, and extracorporeal life-support device.

In addition to the 40-bed general critical department at the UHM, 10 beds are dedicated to surgical CVT patients and this unit is managed by cardiac anesthesiologists/ICU physicians (Figure 5). As part of the department of anesthesiology, the cardiac anesthesia ICU team provides preoperative anesthesia consultations and a 24 h service for the cardiac ICU (six critical care beds and four intermediate care beds) and anesthesia for cardiac, vascular, and thoracic surgery (two operating rooms), as well as for invasive procedures in cardiology (e.g., TEE, cardiac ablation therapy and electrical cardioversion, transcatheter aortic valve implantation, percutaneous mitral valve balloon commissurotomy, and complex percutaneous revascularization).

Covering these clinical duties in addition to administrative and educational tasks requires 9–10 full-time positions, based on a weekly working time of 48 hours. Presently, the anesthesia ICU includes seven certified anesthesiologists/intensivists (80 to 100% position), one cardiologist (40% position), and two to four junior physicians on a six-month internship or fellowship. The shortage of medical personnel is filled by locum positions that are offered over periods of 4 to 12 weeks. The current shortage of anesthesia/ICU staff in Western countries renders the hiring process particularly difficult in overseas Caribbean territories [39].

All cardiac anesthesiologists participate in postgraduate training sessions (on- and off-site) to acquire, maintain, and improve their skills and competencies in echocardiography, ultrasound-guided regional anesthesia, advanced hemodynamic monitoring, and managing sepsis and organ failures. Basic knowledge and practical experience of the nursing staff are enhanced and maintained by attendance at dedicated workshops focused on perioperative physiological monitoring, lung ventilation, point-of-care hemostatic testing, and organ supportive techniques (renal replacement therapy and extracorporeal life support) [40].

Over the past 30 years, the cardiac anesthesia ICU unit has witnessed major changes by the implementation of professional guidelines and innovative approaches to ensure patient safety and successful outcomes following major procedures. The pulmonary artery catheter that was routinely inserted in the early 1990s is now only considered for a few patients with poor ventricular function and/or severe pulmonary hypertension. Transesophageal and transthoracic echocardiography examinations are routinely performed in all cardiac surgical cases and to manage hemodynamically unstable patients [41].

In the ICU, protocol-driven approaches for protective lung ventilation, optimization of systemic oxygen delivery, and prevention of infection, compression wounds, and delirium have been implemented. Clinical rounds are conducted daily with surgeons, weekly with infectious specialists, and on demand with cardiologists. In partnership with the nursing staff, perfusionists, and allied medical specialties, teamwork efforts have been made to conduct scientific research while improving perioperative care, namely by the implementation of an enhanced recovery after surgery (ERAS) program [42] and the provision of advanced hemodynamic monitoring [43] and complementary myocardial protection, particularly in high-risk cardiac patients (i.e., administration of glucose–insulin–potassium) [44].

## 4. Cardiac Surgical Program in Caribbean Territories

### 4.1. Current Situation

Although cardiac surgery started more than 50 years ago in the Caribbean region, access to advanced cardiac interventions varies from island to island and faces difficulties owing to unequal distribution of health care resources and large distances between care providers along with disparities in gross national incomes, insurance coverages, cultures, and languages. Approximately 31 out of 46 million Caribbean residents have direct access to only nine regional cardiac centers (Table 2) [7]. Other patients have no choice but to live with their cardiovascular condition unless they can benefit from surgical treatments delivered by visiting cardiac teams or travel abroad to neighboring or remote countries with specialized cardiac centers. Although several non-profit, nongovernmental organizations attempt to provide high-tech cardiac interventions in some centers (Haiti and Jamaica), the visiting teams perform a small number of procedures, often with a lack of patient education and follow-up as well as less attention paid to training the local health care personnel.

Only three Caribbean hospitals have established residency programs in cardiothoracic surgery (Cuba, Jamaica, and Martinique), but there is still a lack of fellowship training in cardiac anesthesia and critical care.

In the French West Indies, the UHM is expected to cover the need for cardiovascular interventions of about 1.4 million people, whereas in Western countries, there is one cardiac center for a population ranging from 120,000 to 200,000 people [45]. Given the advanced stage and/or number of undiagnosed cardiac diseases, a large number of patients are not referred to cardiac surgery. Noteworthy, the COVID-19 epidemic revealed that ICU capacities in French overseas territories were largely unprepared to face such a crisis and were much lower than average European standards (11.5 ICU beds/100,000 people in Europe vs. 4.5/100,000 in French Guiana and 7.2/100,000 in Martinique and Guadeloupe) [46].

Among Caribbean cardiac centers, the surgical teams from Cuba, Jamaica, Dominican Republic, and Trinidad and Tobago have reported clinical data that confirm the high burden of cardiovascular factors (e.g., hypertension, diabetes mellitus, and kidney disease) and the low numbers of cardiac procedures relative to the total population [47,48,49,50,51].

In Trinidad and Tobago (population of 1.4 million), a permanent cardiac surgical care service was launched in 2006 after monthly visits had been organized since 1993 with a team from the Bristol Heart Institute [48]. In contrast, in Aruba, a high-income country that is part of the Netherlands Antilles (106,000 inhabitants), a cardiac surgical program that started in the early 2000s was interrupted after 6 years owing to a low case load and ensuing disproportionately high costs. Since then, 10% to 14% of the total health care budget is spent on sending patients abroad to Colombia (urgent cases) or the Netherlands (elective cases) to receive cardiac surgical care.

### 4.2. Future Prospects 

Health care models in Caribbean territories must be reinvented to close the gap of unmet needs for invasive cardiac procedures. Setting up a new cardiovascular center or augmenting the capacities of an existing center requires a strong business plan with a comprehensive analysis of key parameters: (1) population (number of potential surgical candidates), (2) insurance health care system with free care or a fee for service reimbursement (public and private), (3) support from the government, insurance companies, and/or charitable foundations to finance the infrastructure and equipment, and (4) availability of qualified and trained human resources in geographically safe and easily accessible areas [52].

Training a complete team (surgeons, anesthesiologists, perfusionists, and nurses) in a foreign academic center is quite expensive and time-consuming and is focused on a few individuals with no guarantee that they will return to their home country.

Expanding the operating capacities of the existing cardiac centers represents a suitable strategy for a hub that should cover the need for advanced cardiovascular treatments and specialized training of health care professionals within the Caribbean region. Financial support from governmental authorities and commitments from health insurance companies and/or charitable foundations are key prerequisites to a successful long-term project. A task force from the EACTS and the World Heart Foundation offers the opportunity to attend educational sessions via the Internet, complemented with on-demand local visits to implement unified standardized approaches in perioperative care [53].

The shortage of medical personnel, particularly cardiac anesthesiologists, could be solved by training young graduates from Caribbean Medical Schools and by providing fellowship programs in cardiothoracic anesthesia and critical care at the UHM and other specialized cardiac centers. Today, postgraduate residency programs on several Caribbean campuses offer a robust curriculum on par with those of North America and Europe with competency-based clinical training supplemented by didactic evidence-based tutorial sessions and certified skills-based workshops conducted in a simulated environment with occasional wet labs [54,55].

Lastly, the gap between clinical duties and academic activities could be addressed by the establishment of a perioperative database to control patient safety and the quality of clinical care, participation in international clinical trials, and the initiation of research programs to tackle the specific risk factors of the Caribbean population. The organization of scientific meetings of cardiac anesthesiologists, intensivists, and surgeons within the Caribbean region would be helpful to share experiences, plan clinical pathways for cardiac patients, and foster the development of research projects.

## 5. Conclusions

Over the last 30 years, the UHM has witnessed remarkable progresses in the development of invasive cardiovascular treatments along with a teambuilding approach involving many health care stake holders (e.g., cardiologists, surgeons, anesthesiologists, emergency physicians, physiotherapist, nurses, and perfusionists).

The application of modern surgical approaches in collaboration with academic institutions abroad has led to improved patient survival and quality of life. Cardiac anesthesiologists have demonstrated perioperative abilities to implement evidence-based clinical care processes and to adapt to work professionally in a complex environment interacting with multiple partners.

The successful development of cardiovascular and thoracic surgical programs in the French West Indies could inspire health authorities in Caribbean territories to pool their resources into specialized surgical hubs linked through a regional health care network involving both hospital and home-based health care workers.

## Figures and Tables

**Table 1:** Geographic variation in the prevalence of cardiovascular diseases.

Pathology	French West Indies and French Guiana	Western Countries (France)
Obesity	23%	16%
Arterial hypertension	23–31%	20%
Diabetes mellitus	12%	5.7%
Sickle cell anemia (homozygous) Sickle cell trait (heterozygous)	1 in 300 to 600 7–8%	Less than 1 in 5000 2.7%
Cardiac amyloidosis	3–5%	Less than 1.5%
Human immunodeficiency virus infection	7–39/100,000	2.3/100,000
Rheumatic heart disease	6.5/100,000	0.9/100,000
Infective endocarditis	19/100,000	17/100,000

**Table 2:** Cardiac surgical care in Caribbean territories.

Territory—Country	Population, n *		Cardiac Surgery	
			**Start Year**	**Type of Program**
Bahamas	415,223		1994	Independent permanent program
Barbados	281,995		1994	Independent permanent program
Cayman Islands—UK	90,000		2014	Independent permanent program
Cuba	11,500,000		1968	Independent permanent programs Cardiac training program
Dominican Republic	11,465,847		n.a.	Local and visiting teams
Jamaica	2,838,235		1994	Local and visiting teams Cardiac training program
Haiti	11,000,000		1996	Visiting teams and patient transfer abroad
Martinique—F	366,981		1988	Independent permanent program Cardiac training program
Guadeloupe—F	395,839			
French Guiana—F	312,121			
Saint Martin—F	32,077			
Puerto Rico—USA	3,260,314		1992	Independent permanent program
Trinidad and Tobago	1,535,1019		1993	Independent permanent program
Anguilla—UK	15,900	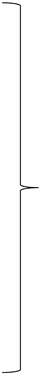		Air lifting patients to cardiac surgical centers in Caribbean territories, Colombia, France, Netherlands, United Kingdom, or United States
Antigua and Barbuda	94,298			
Aruba—NL	106,277			
Belize	410,825			
Caribbean Netherlands—NL	27,148			
Curacao—NL	192,077			
Dominica	73,040			
Grenada	126,184			
Montserrat	4387			
Saint Kitts and Nevis	47,755			
Saint Lucia	180,251			
Sint Maarten	44,222			
Saint Vincent and the Grenadines	103,699			
Turks and Caico Islands—UK	46,062			
Virgin Islands—UK	31,538			
Virgin Islands—USA	98,750			

* n, population number; United Nations Statistics Division, 11 July 2022. Retrieved 2 December 2024. F, France; NL, Netherlands, UK, United Kingdom; USA, United States of America.
